# Data Concatenation, Bayesian Concordance and Coalescent-Based Analyses of the Species Tree for the Rapid Radiation of *Triturus* Newts

**DOI:** 10.1371/journal.pone.0111011

**Published:** 2014-10-22

**Authors:** Ben Wielstra, Jan W. Arntzen, Kristiaan J. van der Gaag, Maciej Pabijan, Wieslaw Babik

**Affiliations:** 1 Naturalis Biodiversity Center, Leiden, The Netherlands; 2 Department of Animal and Plant Sciences, University of Sheffield, Sheffield, United Kingdom; 3 Leiden University Medical Center, Forensic Laboratory for DNA Research, Leiden, The Netherlands; 4 Institute of Environmental Sciences, Jagiellonian University, Kraków, Poland; 5 Institute of Zoology, Jagiellonian University, Kraków, Poland; Field Museum of Natural History, United States of America

## Abstract

The phylogenetic relationships for rapid species radiations are difficult to disentangle. Here we study one such case, namely the genus *Triturus*, which is composed of the marbled and crested newts. We analyze data for 38 genetic markers, positioned in 3-prime untranslated regions of protein-coding genes, obtained with 454 sequencing. Our dataset includes twenty *Triturus* newts and represents all nine species. Bayesian analysis of population structure allocates all individuals to their respective species. The branching patterns obtained by data concatenation, Bayesian concordance analysis and coalescent-based estimations of the species tree differ from one another. The data concatenation based species tree shows high branch support but branching order is considerably affected by allele choice in the case of heterozygotes in the concatenation process. Bayesian concordance analysis expresses the conflict between individual gene trees for part of the *Triturus* species tree as low concordance factors. The coalescent-based species tree is relatively similar to a previously published species tree based upon morphology and full mtDNA and any conflicting internal branches are not highly supported. Our findings reflect high gene tree discordance due to incomplete lineage sorting (possibly aggravated by hybridization) in combination with low information content of the markers employed (as can be expected for relatively recent species radiations). This case study highlights the complexity of resolving rapid radiations and we acknowledge that to convincingly resolve the *Triturus* species tree even more genes will have to be consulted.

## Introduction

The importance of molecular data in biological systematics can hardly be overstated, but potential pitfalls should be considered. In particular, a single gene tree does not necessarily reflect the phylogenetic relationships among species – hereafter referred to as the species tree – as phenomena like incomplete lineage sorting and introgression cloud the pattern of descent [1]. The mitochondrial genome is inherited as a single unit and gives rise to a single gene tree. On the other hand, the nuclear genome, due to its recombining nature, represents a collection of gene trees embedded in the species tree. To distill the species tree, a multitude of nuclear genes should be employed [2]. The progress in next-generation sequencing facilitates the production of large datasets and the use of multilocus datasets in systematics will soon become the norm [3], [4].

The increase in molecular data is followed by advances in analytical methods. It is now realized that combining alignments in a supermatrix that is treated as if it was a single ‘supergene’ can be misleading as this approach ignores the discordance among gene trees in phylogeny reconstruction. High confidence can be appointed to incorrectly inferred evolutionary relationships under data concatenation [3], [5], [6]. Furthermore, choice of allele in the concatenation process in the case of heterozygote marker-individual combinations can lead to widely differing topologies [7], [8]. Gene tree discordance is explicitly incorporated in Bayesian concordance analysis, in which individual gene trees are summarized to provide a ‘concordance factor’ per clade, representing the proportion of gene trees in which clades are present. [9], [10]. Recent coalescent-based methods of species tree estimation take advantage of the information contained in a sample of gene trees more directly by conjointly estimating the species tree and individual gene trees, while explicitly taking into account incomplete lineage sorting as a source of gene tree discordance [2], [11]. Despite these analytical advances, rapid radiations with temporally closely spaced branching events tend to show much incongruence among gene trees and still pose a challenge, even for coalescent-based methods [12], [13]. Reconstructing relationships under such scenarios may be further complicated by ongoing gene flow, especially when it occurs between non-sister taxa [14], [15]. Finally, the intense computational demands for coalescent-based analysis of large data set pose a limiting factor [7].

**Figure 1:**
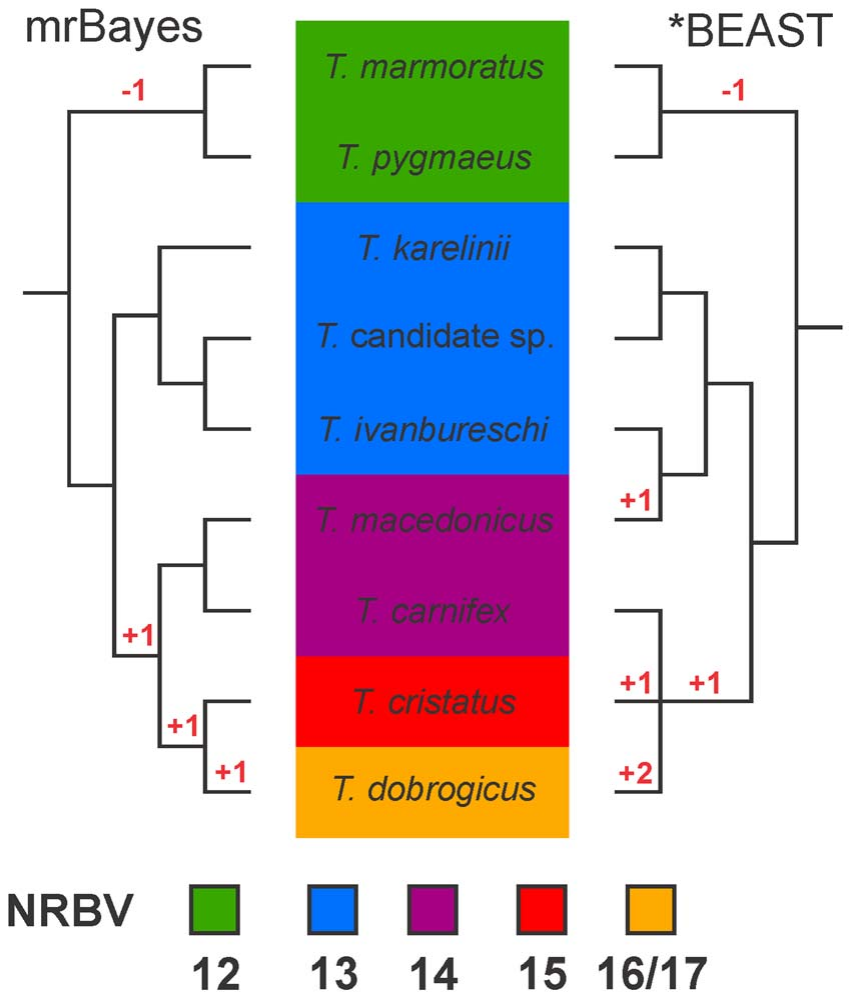
Previously argued phylogenetic hypotheses for the genus *Triturus*. Background colors reflect the variation in the number of rib-bearing pre-sacral vertebrae (NRBV) characterizing the five *Triturus* morphotypes. The two species with a green background are marbled newts and the remaining species are crested newts. The MrBayes phylogeny (left) is based on full mitochondrial genomes [19]. Posterior probabilities for all internal branches are 0.95 or higher. Note that this phylogeny is concordant with the most parsimonious interpretation of the evolution of the number of rib-bearing pre-sacral vertebrae (with the required character state changes noted in red). The *BEAST coalescent-based estimation of the species tree (right) is based on three nuclear introns (adapted from [20]). Posterior probabilities for internal branches are all below 0.95 and only those supported at over 0.75 are shown.

The genus *Triturus* (marbled and crested newts, Amphibia: Salamandridae) provides an example of a rapid radiation and the branching order has proved difficult to resolve [16]. The nine species can be divided into five groups hereafter referred to as morphotypes (Fig. 1; [17]). Morphotypes differ in body built and body built is reflected by the number of rib-bearing pre-sacral vertebrae. Arranged from stocky to slender, with the modal rib-bearing pre-sacral vertebrae count provided between parentheses, these morphotypes are: *T. marmoratus* and *T. pygmaeus* (12), *T. ivanbureschi* (including an as yet unnamed taxon) and *T. karelinii* (13), *T. carnifex* and *T. macedonicus* (14), *T. cristatus* (15) and *T. dobrogicus* (16 or 17) [18]. A full mtDNA dataset yielded a fully bifurcating and highly supported tree in which morphotypes are monophyletic (Fig. 1a; [19]). This tree supports the most parsimonious scenario of character state change in the number of rib-bearing pre-sacral vertebrae (the ancestral state for the number of rib-bearing pre-sacral vertebrae in the family Salamandridae is considered to be 13 [19]). Coalescent-based species tree estimations based on three nuclear markers for all species (Fig. 1b; [20]) and five nuclear markers for a subset of species [21] yielded contrasting results and both deviated from the species tree based on full mtDNA and morphology.

The mitochondrial genome has two characteristics that are particularly important in the setting of a rapid radiation and that make it less susceptible to yield error than a nuclear gene tree. Firstly, lineage sorting occurs faster compared to the nuclear genome due to its fourfold smaller effective population size [1], [22]. Secondly, the number of substitutions accumulated along short internal branches is generally elevated for mtDNA as it is characterized by a comparatively high mutation rate [12], [23]. These advantages, plus the congruency of the full mtDNA phylogeny and morphology, led us to accept the full mtDNA phylogeny over the nuclear phylogenies and we concluded that a wider sampling of the genome is required to test the *Triturus* species tree [19], [20]. We here collect sequences for 38 genetic markers (constituting 13,517 bp in total) for twenty *Triturus* newts. We employ both data concatenation and coalescent-based estimations of the *Triturus* species tree and discuss the discordant outcomes of the analyses in the light of previous attempts to resolve the *Triturus* species tree.

## Material and Methods

### Sampling

**Figure 2:**
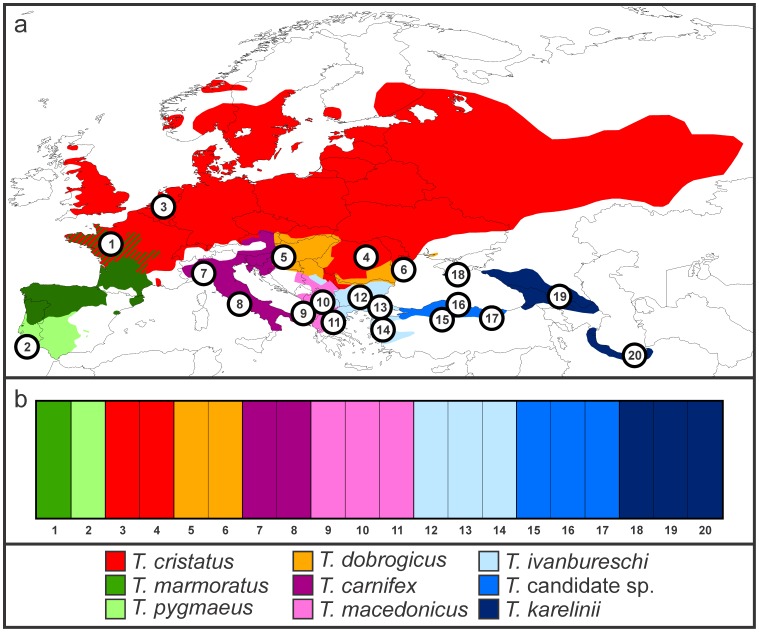
The distribution of the two marbled and seven crested newt species, represented by different colors, and the geographical position of sampled individuals (a). Sampling details can be found in Table 1. BAPS plot showing that each individual is allocated to its respective species (b).

We chose samples from a comprehensive DNA database stored in the collection of Naturalis Biodiversity Center available from an earlier study [24]. This DNA was initially extracted using the DNeasy Tissue Kit (Qiagen) from tail tips taken from animals under anesthesia that were subsequently released back into the wild (a method which does not negatively affect survival [25]). We sampled 2–3 individuals for each of the seven crested newt species and one individual for each of the two marbled newt species. It should be noted that one taxon that we refer to as ‘*Triturus* candidate species’ is yet to be named [26]. We employed a wide geographical spread within species, but our sampling scheme avoided localities positioned close to interspecific hybrid zones, to minimize effects of introgression (Fig. 2; Table 1) [27].

**Table 1:** Details on Triturus sampling.

Population number	Taxon	Sample ID	Locality	N	E
*Marbled newts*					
1	*Triturus marmoratus*	5018*	France: Mayenne	48.252	−0.469
2	*Triturus pygmaeus*	5016	Portugal: Serra de Monchique	37.335	−8.506
*Crested newts*					
3	*Triturus cristatus*	5021*	Netherlands: Heeze	51.393	5.535
4	*Triturus cristatus*	4322	Romania: Sânzieni	46.104	26.128
5	*Triturus dobrogicus*	5065	Hungary: Dráva	46.23	17.06
6	*Triturus dobrogicus*	4801*	Romania: Razboinita	45.222	29.165
7	*Triturus carnifex*	5047	Italy: Donega	44.933	9.233
8	*Triturus carnifex*	5046	Italy: Doganella	41.750	12.783
9	*Triturus macedonicus*	3494	Albania: Bejar	40.429	19.850
10	*Triturus macedonicus*	3424*	Macedonia: Leskoec	40.962	20.885
11	*Triturus macedonicus*	3775	Greece: Kerameia	39.562	22.081
12	*Triturus ivanbureschi*	4732	Bulgaria: Ostar Kamak	41.878	25.853
13	*Triturus ivanbureschi*	2360*	Turkey: Keşan	40.917	26.633
14	*Triturus ivanbureschi*	1820	Turkey: Çan	40.006	26.937
15	*Triturus* candidate species	1901	Turkey: Kalecik	40.077	33.341
16	*Triturus* candidate species	1948	Turkey: Cebeci	41.201	34.036
17	*Triturus* candidate species	2051	Turkey: Şebinkarahisar	40.286	38.126
18	*Triturus karelinii*	2109	Ukraine: Nikita	44.538	34.243
19	*Triturus karelinii*	2226	Georgia: Telavi	41.903	45.475
20	*Triturus karelinii*	2390	Iran: Qu’Am Shahr	36.436	52.803

Population numbers correspond to Fig. 1. Individuals are identified with a code that refers to tail tips/DNA extractions stored at Naturalis Biodiversity Center. Individuals marked with an asterisk were used in the primer testing.

### Sequencing and data preparation

We adapted a protocol initially designed for Ion Torrent next-generation sequencing [28] to be used for the 454 platform. The rationale for choosing this method was that relatively long DNA fragments could be sequenced. We here summarize the protocol followed and refer to ref. [28] for details. Ninety-six transcriptome-based gene models for *Triturus* (compiled following ref. [29]) with long 3′ untranslated regions were identified. To increase the chance that these gene models represent single copy genes, only those that produced unambiguous hits to a single gene when BLASTed against human and *Xenopus* transcripts were selected [30]. For the identified 3′ untranslated regions we designed 96 primer pairs that amplify 400 ± 10 bp (including primers) with BatchPrimer3.1.0 [31] (Dataset S1).

Primer pairs were tested for cross-species-amplification in five *Triturus* individuals spanning the taxonomic width of the genus (Table 1). PCRs were conducted in a total volume of 25 µl, containing 12.5 µl QIAGEN Taq PCR Master Mix, 2.5 µl primer mix (5 µM), 9 µl Milli-Q water and 1 µ of template DNA and our PCR program consisted of denaturation at 95°C for 30 s, 35 cycles of denaturation at 95°C for 30 s, annealing at 55°C for 30 s and extension at 72°C for 45 s, and a final extension at 72°C for 5 min. We checked whether a single product of expected length was amplified for each species by gel electrophoresis using the E-Gel Precast Agarose Gel system (Life Technologies).

Next we amplified the successful markers for all 20 individuals using the same PCR protocol as above in singleplex PCRs. We determined the concentration of each PCR product with the QIAxcel system and pooled all markers per individual equimolar to yield 20 pools. A unique adaptor was ligated to each pool; we used Roche rapid library adaptors RL1–20 (Roche technical bulletin 2010–010). Sequencing was conducted using a quarter of a 454 next-generation sequencing run. Reads were aligned against the transcriptome-based reference and a SNP report was generated in GS Amplicon Variant Analyzer 2.8 (Roche), where SNPs were called if present in at least 20% of reads for marker-individual combinations. We confirmed SNPs and determined how multiple SNPs were linked in individuals containing two alleles for a marker that differed in more than one position by checking the alignments by eye in GS Amplicon Variant Analyzer 2.8.

Sequences were manually aligned in MacClade 4.08 [32]. As missing data can cause spurious results [33], we discarded indels and we filled in missing individual-marker combinations through Sanger sequencing. Models of sequence evolution were determined for each marker with MrModeltest 2 <http://www.abc.se/~nylander/> using the Akaike Information Criterion (Dataset S2) and the number of polymorphic and parsimony informative sites among the haplotypes for each marker was determined with DnaSP v5 [34].

### Data analysis

We conducted a Bayesian analysis of population structure with BAPS v.5.3 [35]. BAPS assigns individuals to distinct gene pools probabilistically, based on multilocus genetic data, where each individual allele is coded as an integer (two gene copies per marker, which may or may not represent the same allelic variant). We let BAPS determine the most probable number of distinct gene pools (*k*) in the data, evaluating k over a 1 ≤ *k* ≤ 20 range, using ten replicates, and tested for admixture among gene pools. We used DISTRUCT [36] to visualize the BAPS results.

We carried out a concatenated phylogenetic analysis with MrBayes 3.2.2 [37] via the CIPRES Science Gateway [38]. To deal with heterozygosity, we created two input files (hereafter input file a and b) and for heterozygote marker-individual combinations we included each allel in one or the other input file, ensuring that each allel was used at least once. Data partitions were unlinked. We conducted two independent four-chain one billion-generation runs per input file, sampled every 100,000 generations, using a heating parameter of 0.2. Tracer v1.6 <http://tree.bio.ed.ac.uk/software/tracer/> was used to check stabilization of overall likelihood within and convergence between runs for each input file. We discarded the first half of generations as burn-in.

We conducted a Bayesian Concordance Analysis using BUCKy 1.4.3 [39]. We created individual gene trees in MrBayes using a single four-chain 1,000,000 generations run, sampled every 1,000 generations. The tree files resulting from MrBayes were summarized using mbsum (distributed as part of the BUCKy package) and the first half of the trees in each tree posterior was discarded as burn-in. The output of mbsum was further processed in BUCKy to create a primary concordance tree with concordance factors for clades. We implemented one cold and three heated chains, used an alpha multiplier of 5 and increased the rate at which chains in the Metropolis-coupled MCMC swap states (every 50 updates) to improve mixing. The *a priori* level of gene tree discordance (α) was set at 1 and 20 with no obvious influence on results; we report concordance factors for the default value of 1. As BUCKy requires a single sequence to represent each marker-individual combination, we followed a similar approach as above to deal with heterozygosity and conducted two replicates, which included either one or the other allele in the case of heterozygote marker-individual combinations.

A coalescent-based estimation of the species tree was performed using *BEAST [40] as implemented in BEAST 1.7 [41]. We applied the lognormal relaxed clock model, the piecewise linear with constant root population function and the Yule speciation model. We used the nine *Triturus* species as operational taxonomical units. We conducted eight independent two billion-generation runs, sampled every 100,000 generations. Tracer v1.6 was used to check for stabilization of overall likelihood. For further analysis we used the three runs that reached the optimum likelihood within the burn-in of one billion generations. These runs were combined and summarized with the LogCombiner 1.7 and TreeAnnotator 1.7 programs distributed with the *BEAST package.

### Data accessibility

The raw 454 reads (in SFF format), the additional sequences obtained with Sanger sequencing (in ABI format), the SNP report produced with GS Amplicon Variant Analyzer 2.8, the alignments (raw, with indels removed, and collapsed into haplotypes), the input files for BAPS, MrBayes, BUCKy and *BEAST, the individual gene trees resulting from MrBayes (created during the BUCKy exercise) and the species tree resulting from the eight *BEAST runs are available under Dryad Digital Repository entry doi:10.5061/dryad.mm81p

## Results

Out of the 96 markers tested, 43 produced a single clear band of expected size on agarose gel for all five tested crested newt species (Dataset S3) and these 43 markers were amplified for all 20 *Triturus* individuals. The 454 run produced 180,120 reads of which 123,674 could be mapped to references. On average we obtained 143.8 reads ± 6.5 (standard error) per marker per individual. Five markers were excluded because they were suspected to represent a multicopy gene (ace), had homopolymer tracts that hampered sequencing (ddx17, trab), or individuals had missing data which we did not manage to complement with Sanger sequencing (dgcr2, rbm15). For the 38 remaining markers, the 19 individual-marker combinations that were missing were sequenced with Sanger sequencing and added to the data set.

Across all *Triturus* species and all 38 markers, the average number of polymorphic sites and the number of parsimony informative sites were 18.0±7.3 (standard error) and 7.6±4.9 and for the crested newts only they were 12.1±5.6 and 4.1±3.7 (Dataset S2). The average number of alleles per marker within individuals ranges from 1.0 to 1.6, with *T. dobrogicus* showing the highest heterozygosity of all species. The genotypes of the twenty *Triturus* individuals can be found in Dataset S4.

BAPS partitioned the twenty *Triturus* individuals into nine groups and each individual clustered with conspecifics with full support (p = 1.0) in the admixture analysis (Fig. 2). We should note that when we forced BAPS to partition the twenty individuals into two groups (given the rational that there is a basic split in *Triturus* between marbled and crested newts) or five groups (given the rational that there are five *Triturus* morphotypes) results slightly deviated from expectation, suggesting limited ability of the program to be used in such a hierarchical manner. Under *k* = 2 the morphotype comprising *T. karelinii*, *T. ivanbureschi* and the *Triturus* candidate species (forthwith the *T. karelinii* morphotype) was distinguished from the other *Triturus* species (including both crested and marbled newt species). Under *k* = 5 the *T. cristatus* and *T. dobrogicus* morphotypes were placed in a single group whereas *T. ivanbureschi* and the *Triturus* candidate species were placed in a separate group from *T. karelinii*.

**Figure 3:**
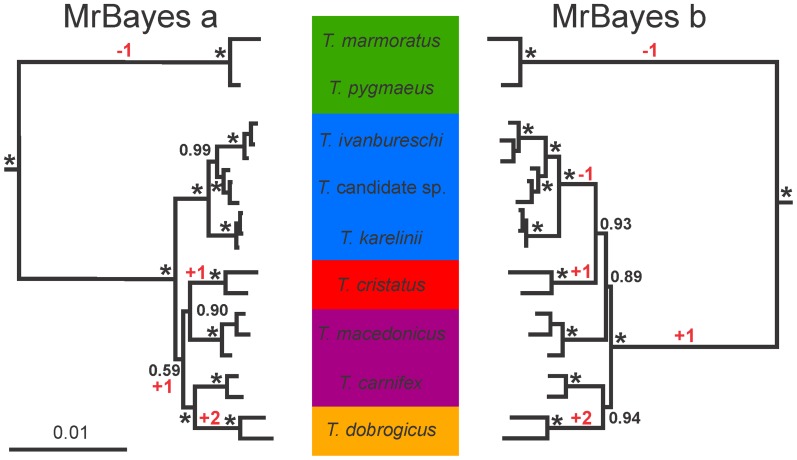
Species trees resulting from the concatenation-based estimation in MrBayes for the genus *Triturus*, based on 38 nuclear markers positioned in 3′ untranslated regions, sequenced for 20 individuals. Internal branches supported with pp = 1.0 are marked with an asterisk. Analyses a and b reflect two different input files used which, for heterozygote marker-individual combinations, include either one or the other allele. The inferred position of the root for *Triturus* is shown. Background colors reflect variation in the number of rib-bearing pre-sacral vertebrae characterizing *Triturus* morphotypes as in Fig. 1 and the character stage changes required are noted in red.

**Figure 4:**
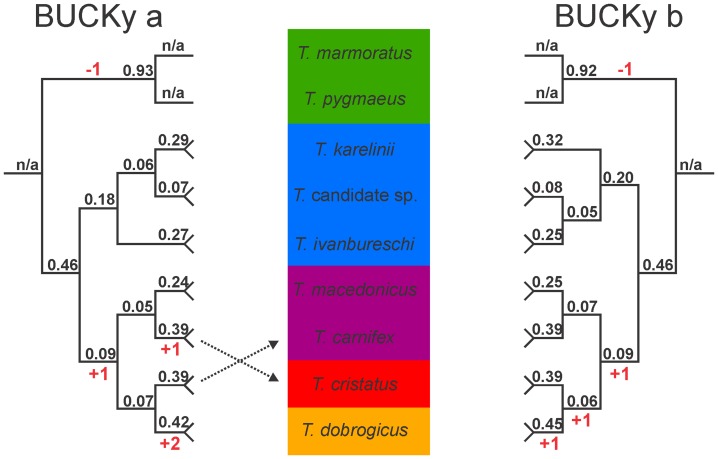
Primary concordance trees resulting from the Bayesian concordance analysis in BUCKy for the genus *Triturus*, based on 38 nuclear markers positioned in 3′ untranslated regions, sequenced for 20 individuals. Support values represent concordance factors, i.e. the proportion of gene trees in which clades are present; n/a is not applicable. Relationships within species are not shown. Analyses a and b reflect two different input files used which, for heterozygote marker-individual combinations, include either one or the other allele. The inferred position of the root for *Triturus* is shown. Background colors reflect variation in the number of rib-bearing pre-sacral vertebrae characterizing *Triturus* morphotypes as in Fig. 1 and the character stage changes required are noted in red.

All species were recovered as monophyletic in the MrBayes concatenated analysis (posterior probability, pp = 1.0; Fig. 3). The topologies resulting from the BUCKy Bayesian concordance analysis recovered all species as monophyletic with relatively high support for the recognized *Triturus* species (concordance factors, CFs ≥ 0.24) compared to the *Triturus* candidate species (CF = 0.07; Fig. 4).

The result of the concatenated analyses (using two different input files together representing all alleles present) with MrBayes (hereafter MrBayes phylogeny) is presented in Fig. 3. The basal dichotomy separates the marbled and crested newt groups with high support (pp = 1.0 for both groups). Additionally, high support for the monophyly of the *T. karelinii* morphotype is found (pp = 1.0) and within the *T. karelinii* morphotype the sister-group relationship of *T. ivanbureschi* and the *Triturus* candidate species has high support (pp = 1.0). Both replicates suggest monophyly of *T. dobrogicus* and *T. carnifex*, albeit support values slightly differ (pp = 1.0 and pp = 0.94). The phylogenetic position of *T. cristatus* and *T. macedonicus* varies between both replicates, although the support values for their placement in both replicates are relatively high (pp = 0.89–0.93).

The result of the Bayesian concordance analysis with BUCKy (hereafter BUCKy phylogeny) is presented in Fig. 4. The topologies of the two replicates differ slightly, but concordance factors for the affected clades are low (<0.1). Support is higher for monophyly of the marbled newts (CF = 0.92 or 0.93 in the two replicates) than for monophyly of the crested newts (CF = 0.46 in both replicates) and support for monophyly of the *T. karelinii* morphotype is lower still (CF = 0.18 or 0.19). Support for relationships among the crested newt morphotypes is low (CFs <0.1). Consensus trees of the posterior tree distributions produced by MrBayes for the individual gene trees are available from Dryad.

**Figure 5:**
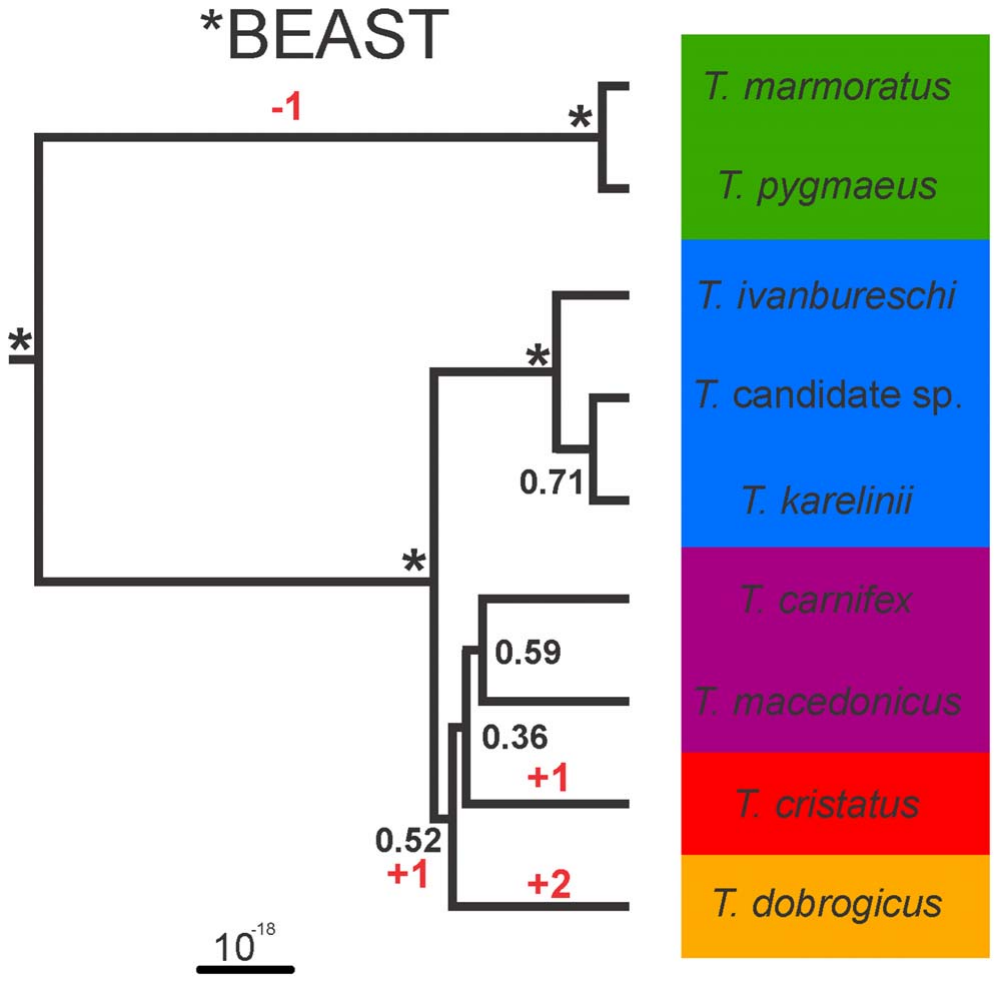
Species trees resulting from the coalescent-based estimation in *BEAST for the genus *Triturus*, based on 38 nuclear markers positioned in 3′ untranslated regions, sequenced for 20 individuals. Internal branches supported with pp = 1.0 are marked with an asterisk. The inferred position of the root for *Triturus* is shown. Background colors reflect variation in the number of rib-bearing pre-sacral vertebrae characterizing *Triturus* morphotypes as in Fig. 1 and the character stage changes required are noted in red.

The result of the coalescent-based analysis with *BEAST (hereafter *BEAST phylogeny) is presented in Fig. 5. This tree is based on three runs; it excludes five runs that plateaued at a lower likelihood value within the burn-in of one billion generations. The individual trees resulting from all eight runs are available on Dryad. Part of the ESS values in the individual analyses, including those runs used to create the consensus tree, were (well) below 200. This finding shows that, even with runs of two billion-generations, stabilization within and convergence between runs did not occur. Considering we conducted eight independent, long runs, this appears inherent to the nature of our dataset. Still the results provide insight into the *Triturus* species tree.

*BEAST consistently supports the monophyly of the marbled newt group, the crested newt group and the *T. karelinii* morphotype (pp = 1.0 in the consensus tree and the individual trees). Relationships among the three members of the *T. karelinii* morphotype are ambiguous, reflected by low support values and various relationships suggested in the individual trees. For the remaining crested newts, support for the sister-group relationship of *T. carnifex* and *T. macedonicus* is low, but it is consistently found, i.e. in the consensus tree and all individual trees. Similarly, all runs find a sister relationship of the *T. carnifex – T. macedonicus* morphotype with *T. cristatus*, albeit again with low support. The phylogenetic placement of *T. dobrogicus* varies between runs, being recovered as either sister to the all crested newts or to the crested newts excluding the *T. karelinii* morphotype (always with low posterior probabilities).

## Discussion

### Distinguishing *Triturus* species with nuclear DNA

With the suite of nuclear DNA markers presented here the various *Triturus* species can successfully be identified. BAPS partitions the dataset into nine gene pools, corresponding to the nine *Triturus* species. Similarly the species were recovered as monophyletic in the data concatenation approach with MrBayes and the Bayesian concordance analysis with BUCKy. This gives us the confidence that possible adverse effects of *a priori* appointing individuals to ‘species’ (e.g. if individuals of more than one species are appointed to a single ‘species’ or individuals of the same species are divided into more than one ‘species’), such as required in the coalescent-based species tree analysis [13], are negligible.

### The different *Triturus* species trees compared

We have a previous estimate of the *Triturus* species tree based on full mtDNA that is in line with the most parsimonious interpretation of evolution of the axial skeleton as reflected by the number of rib-bearing pre-sacral vertebrae, namely four character state changes (Fig. 1a [19]). The topology of the second BUCKy (i.e. the Bayesian concordance analysis) replicate is identical to the full mtDNA phylogeny (Fig. 1 and 4 “Bucky b”). For the other topologies, two branches have to be moved to reconcile them with the topology with full mtDNA and morphology as follows. In the two replicates of the MrBayes phylogeny (i.e. the concatenated analysis; Fig. 3), *T. carnifex* should be moved to form a sister group with *T. macedonicus* and *T. cristatus* should be moved to form a sister group with *T. dobrogicus* (notice that the placement of *T. cristatus* differs between replicates). In the first replicate of the BUCKy phylogeny (“Bucky a” in Fig. 4) the position of *T. carnifex* and *T. cristatus* need to be switched. In the *BEAST phylogeny (i.e. the coalescent-based analysis) *T. cristatus* should be moved to form a sister group with *T. dobrogicus* (Fig. 5). One of the MrBayes phylogenies (“MrBayes b” in Fig. 3) implies six character state changes in the number of rib-bearing pre-sacral vertebrae, whereas the other estimates of the *Triturus* species tree (the second MrBayes replicate, one of the BUCKy replicates and the *BEAST phylogeny; Fig. 3–5) suggests five character state changes, i.e. a less parsimonious interpretation of evolution of the axial skeleton as reflected by the number of rib-bearing pre-sacral vertebrae as compared to the full mtDNA tree.

Most of the internal branches in the two replicates of the MrBayes phylogeny are highly supported. Data concatenation is known to potentially provide high support values for erroneous relationships and in this regard the highly supported topological conflicts between the two replicates, reflecting the effect of allele choice for heterozygote marker-individual combinations in the concatenation process, underline that the high support values for the *Triturus* species tree are indeed misleading [5], [42]. Although consistently supporting the monophyly of marbled and crested newts, relationships among crested newt species differ between the replicates. While the MrBayes phylogeny does recover the *T. karelinii* morphotype to be monophyletic, including the sister relationship of *T. ivanbureschi* and the *Triturus* candidate species (in line with [19], contra [20]; see Fig. 1), it suggests otherwise that the *T. carnifex* – *T. macedonicus* morphotype is non-monophyletic (contra [19], as in [20] but with a different phylogenetic position of the two constituent species; see Fig. 1).

The concordance factors in the two replicates of the BUCKy phylogeny, reflecting the proportion of gene trees that support particular clades, are generally well below one. This reflects a high discordance among individual gene trees. Closer inspection of the individual gene trees (available from Dryad) suggests that most of this discordance can be ascribed to low information content, rather than incomplete lineage sorting, as most gene trees show polytomies. Relatively high support is found for the monophyly of the two main groups in *Triturus*, the marbled and the crested newts. Monophyly for the *T. karelinii* morphotype also shows considerable support, but only one of the two replicates suggests monophyly for the *T. carnifex* – *T. macedonicus* morphotype and support is low. Relationships among morphotypes differ between replicates (of which one is in full agreement with the full mtDNA phylogeny) and concordance factors are low.

In the *BEAST phylogeny monophyly of the two morphotypes that constitute more than one species is not contested. However, support for the *T. carnifex* – *T. macedonicus* morphotype is low. Furthermore, relationships within the *T. karelinii* morphotype differ from the full mtDNA phylogeny (raising doubts concerning the previously assumed sister relationship of the *Triturus* candidate species with *T. ivanbureschi* and supporting its genetic distinctiveness [26]). It should be noted that statistical support for the conflicting internal branches in the *BEAST phylogeny is low, but this is also the case for some of the internal branches that are in line with the full mtDNA topology.

To summarize, all three analyses (data concatenation, Bayesian concordance and coalescent-based) support the monophyly of the marbled newts, the crested newts and the *T. karelinii* morphotype. However, findings regarding the relationships among the different crested newt species are highly ambiguous. It should also be noted that the sister species relationship of *T. cristatus* and *T. dobrogicus*, not found in either the MrBayes or *BEAST phylogeny and in only one of the two BUCKy replicates (with a low CF), is the only internal branch in the full mtDNA tree not supported with pp = 0.95 [19]).

### Interpretation of conflicting *Triturus* species trees

The relative internal branch lengths in the MrBayes and *BEAST phylogenies are (even) shorter than in the full mtDNA phylogeny [19]. Short internal branches highlight the brief time span in which speciation of the four crested newt morphotypes occurred (starting c. 10 million years ago and spanning a period of c. 1.5 million years [19]) and during which there would have been little time for informative substitutions to become fixed among the different taxa [12]. The variability of the markers employed is indeed low (Dataset S2), but the ambiguity in the branching order and the low support (for the BUCKy and *BEAST phylogeny) could also reflect a hard polytomy (i.e. a simultaneous split of more than one species) underlying the radiation of *Triturus* [16]. *Triturus* newts hybridize at the contact zones [43] and the position of hybrid zones has shifted over time [44]–[46]. The lack of phylogenetic signal might be aggravated by gene flow between sister taxa (shortening internal branch lengths) or non-sister taxa (increasing support for the clustering of non-sister taxa) [14], [15].

Reconstructing the phylogenetic relationships of rapid radiations remains a challenging task as the risk of gene trees deviating from their overarching species tree is high [47], [48]. We reiterate the conclusion that data concatenation is likely to lead to high support for erroneous phylogenies and is strongly affected by allele choice in the case of heterozygosity and results from such an analysis should be interpreted with caution [5], [7]. Bayesian concordance analysis of rapid radiations will inherently result in a poorly supported tree as gene tree discordance is to be expected [7]. The development of coalescent-based methods to estimate species trees is an important step forward in phylogenetic inference of rapid radiations [42], [49]. Compared to data concatenation, coalescent-based estimations of the species tree are more robust to recovering erroneous relationships due to incomplete lineage sorting [7], [12] or persistent interspecific gene flow [50]. It follows that, for rapid radiations in particular, coalescent-based estimations of the species tree would bring us closer to the truth than data concatenation [12], [13]. Although increasing the number of base pairs sequenced will assist in harvesting informative substitution over short internal branches, the application of coalescent-based species tree estimation methods is still hampered by computational limitations [3], [8], [12]. Hence, for large datasets Bayesian concordance analysis will be the more tractable option for some time to come.

Based on theoretical grounds, the coalescent-based estimation of the *Triturus* species tree would be expected to be more reliable than the concatenation-based estimation. However, due to the low information content of the markers, in combination with the rapidness of the *Triturus* radiation, the coalescent-based estimation does not manage to confidently resolve the complete *Triturus* species tree. Hence we acknowledge that more loci and individuals are required to arrive at a firm conclusion. The coalescent-based estimation shows a relatively high agreement with full mtDNA and morphological data, but as full mtDNA reflects only a single gene tree and ecology could drive homoplastic evolution, we have to consider that this agreement is potentially misleading. The genus *Triturus* highlights the difficulty of resolving the branching order of a rapid radiation, in which adding more data does not necessarily increases confidence in the obtained phylogeny. We consider *Triturus* to be a suitable study system to explore the effects lineage sorting, gene flow and mutation rate at the interface of a rapid radiation and phylogenetic reconstruction.

## Supporting Information

Dataset S1 The 96 transcriptome-based gene models used for primer development, primers and reference sequences.
(XLSX)

Dataset S2 Models of sequence evolution for and variation in the 38 markers.
(XLSX)

Dataset S3 Testing of 96 primer pairs for cross-amplification for the genus *Triturus*.
(XLSX)

Dataset S4 Distribution of alleles per marker across the twenty *Triturus* individuals.
(XLSX)
